# Comparison of Product Features and Clinical Trial Designs for the DTx Products with the Indication of Insomnia Authorized by Regulatory Authorities

**DOI:** 10.1007/s43441-024-00684-9

**Published:** 2024-09-21

**Authors:** Takashi Hosono, Yuki Niwa, Masuo Kondoh

**Affiliations:** 1 Graduate School of Pharmaceutical Sciences, Osaka University, 1-6 Yamadaoka, Suita, 565-0871 Osaka Japan; 2 Clinical Research, R&D, NS Pharma Inc, Paramus, NJ 07652 USA

**Keywords:** Digital therapeutics, DTx, Insomnia, Sleep disorder, Clinical trials

## Abstract

**Background:**

Digital therapeutics (DTx) have attracted attention as the substitutes or add-ons to conventional pharmacotherapy and the number of DTx products authorized with the regulatory reviews of the clinical evidence is increasing. Insomnia is one of the major targets of the DTx due to the benefit from cognitive behavioral interventions and several products have been launched in the market with regulatory reviews. However, common features of the products and the clinical evidence required by each regulatory agency have not been investigated.

**Methods:**

In this study, we identified the DTx products with the primary indication of insomnia authorized with regulatory reviews of clinical evidence by literature and website searches, and investigated the common features of the products and of the study designs for the pivotal clinical trials.

**Results:**

The total of 6 DTx products were identified. The components of cognitive behavioral therapy for insomnia (CBT-I) were identified as common features of the products. All the pivotal clinical trials were randomized, parallel-group, blind studies against insomnia patients. No products have been authorized in multiple countries regardless of the similarity of the features of the products and of the study designs for the pivotal clinical trials.

**Conclusions:**

Our study revealed that the components of CBT-I and gold standard design in pivotal clinical trials were adopted in all the DTx products for insomnia authorized with reviews of clinical evidence. At the same time, our findings suggest the needs of internationally harmonized regulatory review and authorization system to facilitate the early patient access to the promising DTx products.

**Supplementary Information:**

The online version contains supplementary material available at 10.1007/s43441-024-00684-9.

## Introduction

In recent years, digital Therapeutics (DTx) have attracted the attention as the substitutes or add-ons to conventional pharmacotherapy. According to the definition by International Organization for Standardization (ISO), DTx are defined as health software intended to treat or alleviate a disease, disorder, condition, or injury by generating and delivering a medical intervention that has a demonstrable positive therapeutic impact on a patient [1]. DTx are evidence-based therapeutic interventions to specific medical conditions and the effectiveness of use must be proven through systematic clinical trials. DTx must be also reviewed and authorized by regulatory authorities as required to support product claims of risk, efficacy and intended use [2, 3].

In many countries, DTx are regulated through the similar regulatory framework with medical devices as part of Software as a Medical Device (SaMD), which is defined as software intended to be used for one or more medical purpose(s) that perform(s) these purposes without being part of hardware medical device by International Medical Device Regulators Forum (IMDRF) [4]. SaMD is also defined under the name of Medical Device Software (MDSW) in European Medical Device Regulation (EU MDR) as software that is intended to be used, alone or in combination, for a purpose as specified in the definition of a medical device [5]. Digital Therapeutic Alliance (DTA) mentions it is important to note that not all DTx products qualify as SaMD, and not all SaMD products qualify as a digital therapeutic [6].

Based on the level of control necessary to assure the safety and effectiveness set by each regulatory agency, some DTx products are authorized with the regulatory reviews of clinical trial data while others need only notification, registration or self-declaration of conformity to the regulation in each country [7]. For the regulatory reviews, DTx are usually exempt from non-clinical studies and the clinical evidence from optional pilot trials and pivotal clinical trials are required likewise the development of medical devices [8]. Some DTx products authorized with the regulatory reviews require clinician initiation and oversight, and they are called prescription DTx (PDTx) [2].

Some regulatory authorities have recently implemented the policies which promote the development of SaMD and DTx. In the US, Food and Drug Administration (FDA) launched the Software Precertification Pilot program to foster innovative medical devices software in 2017 and completed the pilot program in 2022 [9]. The Digital Health Center of Excellence was also established at the Center for Devices and Radiological Health in 2020 [10]. In the UK, there is no separate definition for DTx, but National Health Service (NHS) has continued to support the development of apps, digitally-enabled models of therapy and online resources to support good mental health [11]. In Germany, the Digital Health Care Act introduced an innovative process for Digital Health Applications (DiGA) in 2019. All statutory health insurance companies reimburse DiGA in the DiGA directory. The fast-track process enables manufacturers to demonstrate one or more positive healthcare effects by means of a scientific study within 12 months after the provisional inclusion in the directory [12]. In France, prise en charge anticipée numérique (PECAN) fast track offers a one-year transitional reimbursement for eligible DTx products [13]. In Belgium, the certified mobile health applications are listed in mHealthBelgium platform and reimbursed in accordance with the level of the validation pyramid [14]. In Japan, the Digital Transformation Action Strategies in Healthcare (DASH) for SaMD and DASH for SaMD 2 were implemented in 2020 and in 2023 respectively aiming to promote the timely patient access to innovative SaMD [15, 16]. In South Korea, the definition of DTx and the documents required to be submitted when obtaining DTx approval in the Korean regulatory system were specified in the guidelines published in 2020 [17].

With the advancement of digital technology and the implementation of regulatory policies to promote the development and the patient access, the number of clinical trials and the products authorized with the regulatory reviews are also increasing in the world [2, 18]. Regardless of the increase of the number of clinical trials and authorized DTx products in the world, not many DTx products have been authorized in multiple countries [2]. Bridging clinical trials to extrapolate foreign clinical data into new regions and multiregional clinical trials (MRCTs) have been widely used for the development of pharmacotherapeutic new drugs in accordance with the international council for harmonization of technical requirements for pharmaceuticals for human use (ICH) E5 and E17 guidelines [19, 20]. However, the similar approaches have not been fully utilized in the development of medical devices including DTx. Consequently, timely patient access to promising DTx products has not been achieved in the world.

The therapeutic areas of DTx are diverse, but psychiatric disorders are one of the major targets because of the benefit from cognitive behavioral interventions [2]. In addition to anxiety disorders, depression, substance use disorder, post-traumatic stress disorder, etc., Insomnia is one of the main psychiatric disorders and also the major symptoms of the other psychiatric disorders [21]. Cognitive behavioral therapy for insomnia (CBT-I) is a multi-component treatment which includes education, cognitive and behavioral interventions, and is recommended as first-line treatment for insomnia in many clinical guidelines [22–24]. However, the disadvantages of face-to-face CBT-I are low accessibility and high cost, and CBT-I through DTx is considered as an ideal alternative [25, 26]. Multiple DTx products for insomnia including CBT-I have been launched in the market with the regulatory reviews and in development processes, but the common features of the products and the clinical evidence required by each regulatory agency have not been investigated.

The objective of our study was to identify the DTx products with the primary indication of insomnia authorized with regulatory reviews of clinical evidence, and to investigate the common requirements for the regulatory authorizations by comparing the features of the products and the study designs of the pivotal clinical trials in order to facilitate the precise and efficient development, leading to early patient access to the promising DTx products for insomnia.

## Materials and Methods

### Search Strategy

The DTx products with the primary indication of insomnia which have been authorized with the regulatory reviews of the efficacy and the safety were identified in the following way.

The total of 4 databases, including PubMed, Web of Science, Science Direct and Google Scholar were systematically searched with the keywords: (“digital therapeutic” OR “digital therapeutics” OR “DTx”) AND (“sleep disorder” OR “sleep disorders” OR “insomnia”) in December 2023. The search language was limited to English. After excluding the duplicates and the records before 2010 automatically on the reference management software, non-English records, non-journal records, non-DTx related records, records only with the other indications than insomnia/sleep disorder, and non-product specified records were excluded by the review of the titles, the abstracts and the methods of the records.

The DTx products with the primary indication of insomnia were also searched in the product library of 3 websites: Digital Therapeutics Alliance [27], DiGA directory [28] and mHealthBelgium [29]. The records with the primary indication of other indications than insomnia/sleep disorder were excluded by the review of the titles and abstracts of the records.

Next, the DTx products with the primary indication of insomnia were identified in the screened records with the full-text review. After removing the duplicated products, the DTx products authorized with the regulatory reviews of clinical evidence were identified by checking the websites of regulatory authorities including health technology assessment (HTA) bodies (Supplementary Table 1), their review reports (Supplementary Table 2), the related scientific literatures and the product homepages. The indications, the regulatory classifications, the authorization year, the necessity of prescription, the prices and the patients’ copay were also checked with the same sources.

In our study, the DTx products that have not been authorized by any regulatory authorities were excluded. Those included wellness apps without authorizations by regulatory authorities. Also, the DTx products authorized by regulatory authorities without regulatory reviews of the clinical trial data were excluded. Those includes registration-only and declaration-only products (e.g. Class I medical devices in many countries including the products only with CE-mark I in EU and UK), and temporarily or provisionally approved products without regulatory reviews of clinical trial data (e.g. products distributed in the US through the FDA enforcement excretion under the COVID-19 and the products provisionally listed in DiGA directory, the positive healthcare effects through a scientific study of which will be reviewed later). The products permanently listed in DiGA directory after the review of positive healthcare effects by BfArM were included in our study.

### Comparison of Features of DTx Products and the Pivotal Clinical Trials

The features of the identified products were examined in the product manuals issued by manufacturers and the related scientific articles. In addition to the type of platforms and the languages the products covered, the presence of the components of CBT-I including sleep diary, sleep restriction therapy, stimulus control therapy, sleep hygiene, cognitive restructuring, muscle relaxation, relapse prevention and other unique features in each product were examined.

The pivotal clinical trial and the study design of each DTx product were identified mainly in the review report by regulatory authorities (Supplementary Table 2). When the review reports were not published by regulatory authorities, the information identified in the clinical trial registration system (Supplementary Table 3) and the related scientific articles were judged as the one reviewed and approved by regulatory authorities. The information of target population, study design, sample size, control, treatment period, primary endpoints and study sites were examined. In our study, we focused on pivotal clinical trials for the regulatory authorization although some pilot studies and post-marketing studies have been conducted in some products. When there were some clinical trials which were equivalent to pivotal clinical trials in one product, the first one in time was adopted in our study.

## Results

### Products Identified by Database and Website Search

**Fig. 1:**
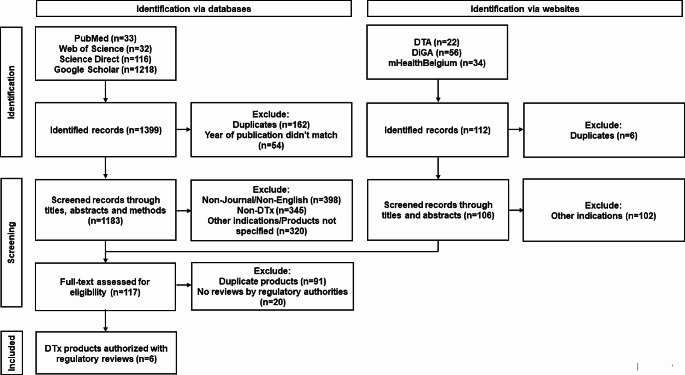
Flow diagram of product identification

As shown in the flow diagram (Figs. 1), 1399 records were identified potentially relevant. Following screening with titles, abstracts, and methods and full-text review, 117 records which included DTx products with the primary indication of insomnia were identified. After the duplicates and the products without regulatory reviews by regulatory authorities in the records were removed, the total of 6 products were identified (Table 1).

**Table 1:** Products identified by database and website search

Product	Manufacture	Primary Indication	Regulatory Agency	Classification	Authorization Year	Prescription	Price (Patients’ copay)
Somryst^®^	Pear Therapeutics (Product acquired by Nox Health)	Chronic Insomnia	FDA (US)	Class II, 510(k)	2020	Required	$899 ($100-$899)
Sleepio^®^	Big Health	Insomnia	NICE (UK)	Recommendation	2022	Not required	£45 (£0 - £45)
Somnio^®^	mementor DE GmbH (Company acquired by Resmed)	Insomnia	BfArM (DE)	Permanently listed in DiGA	2022	Required	€224.99 (Free)
SUSMED Med CBT-I^®^	SUSMED, Inc.	Insomnia	PMDA (JP)	Class II	2023	Required	TBD
Somzz^®^	AIMMED co., Ltd.	Insomnia	MFDS (KR)	Innovative medical device	2023	Required	TBD
WELT-I^®^	WELT Corporation	Insomnia	MFDS (KR)	Innovative medical device	2023	Required	TBD

Abbreviations: BfArM: Bundesinstitut für Arzneimittel und Medizinprodukte; FDA: Food and Drug Administration; MFDS: Ministry of Food and Drug Safety; NICE: National Institute for Health and Care Excellence; PMDA: Pharmaceuticals and Medical Devices Agency

Somryst^®^, previously called SHUTi was cleared by FDA as 510 (k) Class II medical device in 2020 under Software Precertification Pilot program [9]. Somryst^®^ has not been authorized in any other countries outside the US. The indication of Somryst^®^ is limited to chronic insomnia which is different from the other 5 identified products [30]. The prescription is needed to start Somryst^®^. The price is $899, and the patients’ copay depends on the insurance plan [31].

Sleepio^®^ received a CE mark I in 2018 and was recommended by the National Institute for Health and Care Excellence (NICE) in 2022 [32], but the product had been on the market much before in 2013. Sleepio^®^ has also been distributed in the US market under the FDA enforcement excretion under the COVID-19, but has not been regulatory cleared by FDA [33]. Another unique characteristic of Sleepio^®^ is this is not a PDTx. The price of Sleepio^®^ is £45 (excluding VAT), and the insomnia patients who live in England, Scotland and people with or beyond cancer in some selected areas can start Sleepio^®^ free on NHS [34, 35].

Somnio^®^ is the only DTx product for insomnia permanently listed in DiGA directory in 2022 [36]. Somnio^®^ has not been authorized with regulatory reviews in any other countries rather than Germany. Somnio^®^ is a PDTx and the price is €224.99, and the patients can start the program without any out-of-pocket expenses for the products listed in DiGA directory [36, 37].

SUSMED Med CBT-I^®^ is the PDTx product for insomnia authorized only in Japan after the regulatory review by Pharmaceuticals and Medical Devices Agency (PMDA) as Class II medical device in 2023 [38]. The price of medical devices usable in insurance-covered healthcare, is specified by Minister of health, Labor and Welfare (MHLW) in Japan and the price of SUSMED Med CBT-I^®^ has not been determined.

Somzz^®^ is the first DTx product approved in February 2023 by Ministry of Food and Drug Safety (MFDS) in accordance with the guidelines on review and approval of digital therapeutics issued in 2020 [17, 39]. WELT-I^®^ was also approved by MFDS 2 months later [40, 41]. Both products were PDTx authorized only in South Korea and the prices have not been determined yet.

### Features of Identified DTx Products

The features of 6 products were summarized (Table 2). The features of all the products were very similar regardless of the differences of the manufactures and of the authorized countries. Especially, all the identified products had the core components of CBT-I such as sleep restriction, stimulus control, sleep hygiene and cognitive restructuring [42–47]. Some products had additional CBT-I related features. Sleepio^®^ and Somnio^®^ had web applications in addition to mobile applications as platforms. Some products had the functions of muscle relaxation, relapse prevention and some unique functions. On the other hand, only Somnio^®^ covered multiple languages and other products covered only the official language in the authorized countries.

**Table 2:** Features of identified DTx products

Product	Platform	Language	Sleep diary	Sleep restriction	Stimulus control	Sleep hygiene	Cognitive restructuring	Muscle relaxation	Relapse prevention	Other unique functions
Somryst^®^	Mobile	English	Yes	Yes	Yes	Yes	Yes	No	Yes	Clinician dashboard
Sleepio^®^	Mobile, Web	English	Yes	Yes	Yes	Yes	Yes	Yes	No	Paradoxical Intention, Online community, Import of sleep data through FitBit
Somnio^®^	Mobile, Web	English, German, French	Yes	Yes	Yes	Yes	Yes	Yes	No	Import of sleep data through FitBit
SUSMED Med CBT-I^®^	Mobile	Japanese	Yes	Yes	Yes	Yes	Yes	Yes	No	NA
Somzz^®^	Mobile	Korean	Yes	Yes	Yes	Yes	Yes	Yes	Yes	Real-time feedback
WELT-I^®^	Mobile	Korean	Yes	Yes	Yes	Yes	Yes	Yes	No	Cognitive intervention through Chatbot

### Study Design of Pivotal Clinical Trials

The study design of pivotal clinical trial for each product was summarized (Table 3). In addition to the similarity of the features of the products, the study designs of the pivotal clinical trials were also similar among the identified products [45–50].

**Table 3:** Study designs of pivotal clinical trials for identified DTx products

Product	Clinical Trial Number (Study start year)	MRCT (Single or Multiple sites)	Target Population	Randomization/Design	Blinding	Control	Sample size	Treatment period (Follow-up)	Primary endpoints
Somryst^®^	NCT:01438697 (2011)	No (Single site in US)	Patients defined by own criteria*	Randomized/Parallel-group	Single-blind (participant)	Online patient education	303	9 weeks (up to 12 months)	ISI, SOL, WASO
Sleepio^®^	ISRCTN:44615689 (2011)	No (Single site in UK)	Patients diagnosed with DSM-5	Randomized/Parallel-group	Single-blind (participant)	Online imagery relief therapy, TAU	164	6 weeks (up to 14 weeks)	SE
Somnio^®^	NCT:02629913 (2016)	No (Single site in CH)	Patients diagnosed with DSM-5	Randomized/Parallel-group	Single-blind (interviewer)	Waitlist	56	6 weeks (up to 12 months)	ISI
SUSMED Med CBT-I^®^	JRCT:2032210071 (2021)	No (Multiple sites in JP)	Patients diagnosed with ICSD-3	Randomized/Parallel-group	Double-blind	Sham app	175	8 weeks (up to 10 weeks)	AIS
Somzz^®^	CRIS:KCT0007292 (2022)	No (Multiple sites in KR)	Patients diagnosed with ICSD-3	Randomized/Parallel-group	Single-blind (participant)	Sham app	98	6 weeks	ISI, SE, SOL, WASO, TST, DBAS-16, PHQ-9, GAD-7, FSS, ESS, SF-36, WPAI: SHP, EQ-5D-5L
WELT-I^®^	NCT:05809544 (2022)	No (Single site in KR)	Patients diagnosed with DSM-5	Randomized/Parallel-group	Double-blind	Sham app	68	6 weeks (up to 7 weeks)	SE

Abbreviations: AIS: Athens Insomnia Scale; DBAS-16: Dysfunctional Beliefs and Attitudes about Sleep Scale-16; DSM-5: Diagnostic and Statistical Manual of Mental Disorders, Fifth Edition; EQ-5D-5L: EuroQol-5D-5L; ESS: Epworth Sleepiness Scale; FSS: Fatigue Severity Scale; GAD-7: General Anxiety Disorder-7: ICSD-3: International Classification of Sleep Disorders- Third Edition; ISI: Insomnia Severity Index; PHQ-9: Patient Health Questionnaire-9; SF-36: 36-Item Short Form Survey; TAU: Treatment as usual;

* (1) required more than 30 min to fall asleep at the beginning of the night or more than 30 min of time awake after initially falling asleep for at least 3 nights per week for at least 6 months, (2) total sleep time averaged 6.5 h or less, and (3) manifested sleep disturbances (or associated daytime symptoms) causing significant distress or impairment in social, occupational, or other areas of functioning

All the pivotal clinical trials were randomized, parallel-group, blind studies against insomnia patients. The patients were screened by international diagnostic criteria for insomnia such as Diagnostic Statistical Manual of Mental Disorder, Fifth Edition (DSM-5) and International Classification of Sleep Disorders - Third Edition (ICSD-3) except for Somryst^®^ which defined chronic insomnia patients by its own criteria. In the pivotal clinical trials for SUSMED Med CBT-I^®^, Somzz^®^ and WELT-I^®^, sham apps which excluded the clinically active components of CBT-I from the products were used as comparators. The treatment periods of the pivotal clinical trials were 6–9 weeks, but the long-term persistence of the efficacy was also investigated in the pivotal clinical trials in Somryst^®^ and Somnio^®^ (up to 12 months). The sample size varied in each pivotal clinical trials ranging from 56 to 303, but the size to investigate statistical significance against comparators were set in each study. The similar primary endpoints used in pharmacotherapy and cognitive therapy for insomnia such as insomnia severity index (ISI), sleep efficiency (SE), sleep onset latency (SOL) and wake after sleep onset (WASO) were adopted as the primary endpoints of the pivotal clinical trials. No MRCTs were conducted in any of the pivotal clinical trials of the identified products although some pivotal clinical trials were multi-centered trials in one country. The patients living in multi-regions in Switzerland, Austria, and Germany participated in the pivotal clinical trial of Somnio^®^, but this was single-center clinical trial.

## Discussion

### Similarities of Product Features and Study Designs of Pivotal Clinical Trials

Our study revealed that there were a lot of similarities of the features of DTx products for insomnia authorized with the regulatory reviews regardless of the differences of the authorized countries. Especially, all the products had the components of CBT-I which is recommended as the first-line treatments in many therapeutic guidelines for insomnia treatment. Including the components of the treatments recommended in therapeutic guidelines such as CBT-I in DTx products is suggested for the authorization by regulatory authorities.

Our study also revealed that gold standard clinical trial design was adopted in the pivotal clinical trials of the DTx products for insomnia authorized with regulatory review of clinical evidence. All the pivotal clinical trials were randomized, parallel-group, blind studies against insomnia patients which could investigate the statistical significance against comparators for the major efficacy endpoints of insomnia treatment. Investigating the long-term persistency in the pivotal clinical trials is also worthwhile. Those characteristics required in the pivotal clinical trials are consistent between DTx and pharmacotherapies [51]. Blinding by setting digital sham or sham app as comparator is also effective for the clinical trials for DTx, but the careful consideration and development are critical due to the presence of potential therapeutic effects arising from engagement of digital intervention [51, 52]. Sham apps without CBT-I were adopted as comparators in 3 out of the 6 pivotal clinical trials conducted relatively recently [45–47].

### Potential Futures of Clinical Trials and Regulatory Authorizations for DTx

There were no DTx products for insomnia authorized in multiple countries with regulatory reviews of the efficacy and the safety through data bridging or MRCTs regardless of the similarity of the features of the products and of the study designs of pivotal clinical trials. This is a very different status compared to the development of new drugs that a lot of products have been approved by regulatory authorities in several regions by utilizing data bridging and MRCTs in accordance with ICH E5 and E17 guidance. Decentralized clinical trials (DCTs) in the use of remote data that are electrically transmitted from the participants were rapidly introduced in the clinical trials for traditional pharmacotherapies during the COVID-19 [53]. Most DTx products are provided by mobile applications and smart devices, and the characteristics of DTx products must be beneficial to facilitate the clinical trials such as DCTs and MRCTs.

The small company size of most DTx manufacturers may also have restricted the countries and regions where they develop their DTx products. The recent increase of collaboration between DTx manufactures and larger pharmaceutical or medical device companies may lead to simultaneous global development and regulatory authorizations in multiple regions in the future [54].

### Hurdles for Efficient and Precise Development of DTx Products

At the same time, careful considerations are also required for the clinical evaluation of DTx products from the aspects of unique characteristics of DTx and the difference from traditional pharmacotherapies [2]. DTx is intrinsically cognitive and behavioral interventions that receive the impacts of sociocultural and cognitive abilities of patients. The product features including user interfaces need to be adjusted by considering the factors when we conduct MRCTs. The appropriate user training of the products in accordance with the IT literacy of the patients is also needed to keep the quality of the clinical trials including the patient engagement and adherence rate. One more unique hurdle of the MRCTs for DTx is language barriers. The translation and the validation of the product contents are critical for multilingual clinical trials for DTx. Our study revealed the status that all the identified products except for Somnio^®^ cover only the official language in the country where the product was authorized. Part of the reasons might be most DTx manufactures were not traditional big pharmaceutical or medical device companies which were familiar with global development. The knowledge and experiences of electronic Patient-Reported Outcome (ePRO) which have been increasingly adopted in the clinical development for pharmacotherapies might be beneficial in order to overcome the sociocultural, IT literacy and language hurdles. ePRO data are usually collected through special devices in a decentralized manner, and cross-cultural translation has been adopted in a lot of MRCTs [55].

### Regulatory Policies and Reimburse System

New regulatory frameworks, including action plans, programs, guidelines, and departments, have been introduced in some regulatory authorities to promote DTx development [9–17, 56, 57]. However, their international harmonizations have been delay. Although it is difficult to suggest an ideal harmonized regulatory approval pathways from the results of our study, the international framework for the mutual acceptance and evaluation of clinical evidence generated from data bridging studies and MRCTs are required to facilitate the development of DTx products. In addition, the rigid reimbursement system for DTx products is also needed. Pear Therapeutics, the manufacture of Somyrst^®^, went bankruptcy in 2023 due to the limited application to national or private insurance in the US and the limitations in profit generation [58]. SUSMED Inc., the manufacture of SUSMED Med CBT-I^®^ recently withdrew the application of national health insurance coverage along with CBT-I was not covered by national health insurance in Japan [59]. After the regulatory authorization by MFDS, AIMMED co., Ltd. and WELT Corporation which were the manufactures of Somzz^®^ and WELT-I^®^, respectively were requested to prepare for the project with selected hospitals to accumulate the real-world evidence before the nation-wide expansion of the products by the National Evidence-based Healthcare Collaborating Agency (NECA) in South Korea [60].

### Limitation of our Study

There are several limitations in our study. First, we excluded the DTx products which have been authorized without the regulatory reviews of the efficacy and the safety through clinical trials. Those include the products classified in Class I medical devices and temporarily or provisionally approved products by each regulatory agency. Second, we focused on the first pivotal clinical trials for the regulatory authorization in each product. Several products such as Somryst^®^ and Sleepio^®^ have multiple post-marketing clinical trial data. Finally, we didn’t consider the differences of sociocultural aspect and IT literacy in each country that might have impacted on the clinical trials for DTx products in our study.

## Conclusion

Our study revealed that the components of CBT-I and gold standard study design in pivotal clinical trials were adopted in all the DTx products for insomnia authorized with reviews of clinical evidence. At the same time, our findings also suggest that the need of internationally harmonized regulatory review and authorization system. We believe that our study will contribute to the efficient and precise development of DTx products, leading to the early access by the patients to the promising products.

## Electronic Supplementary Material

Below is the link to the electronic supplementary material.

Supplementary Material 1

## Data Availability

No datasets were generated or analysed during the current study.
