# Supplementary material for: Comparison of Product Features and Clinical Trial Designs for the DTx Products with the Indication of Insomnia Authorized by Regulatory Authorities
Source: Ther Innov Regul Sci. 2024 Sep 21;58(6):1138–47. doi: 10.1007/s43441-024-00684-9 (PMC11530488; doi:10.1007/s43441-024-00684-9)
Supplement: Supplementary file 1 — Supplementary Material 1 [file 43441_2024_684_MOESM1_ESM.docx]

**Supplementary table 1 A list of regulatory authorities**

| **Regulatory authority (Country)** | **Link** |
| --- | --- |
| Therapeutic Goods Administration (AU) | <https://www.tga.gov.au/resources/artg> |
| Federal Agency for Medicines and Health Products (BE) | <https://www.famhp.be/en> |
| mHealthBelgium (BE) | <https://mhealthbelgium.be/> |
| Brazilian Health Regulatory Agency (BR) | <https://www.gov.br/anvisa/pt-br/english> |
| Health Canada (CA) | <https://www.canada.ca/en/health-canada.html> |
| National Medical Products Administration (CN) | <https://english.nmpa.gov.cn/index.html> |
| EUDMAED database (EU) | <https://ec.europa.eu/tools/eudamed/#/screen/home> |
| National Agency for the Safety of Medicines and Health Products (FR) | <https://ansm.sante.fr/> |
| PECAN (FR) | <https://gnius.esante.gouv.fr/en/early-access-reimbursement-digital-devices-pecan> |
| Federal Institute for Drugs and Medical Devices (DE) | <https://www.bfarm.de/EN/Home/_node.html> |
| DiGA directory (DE) | <https://diga.bfarm.de/de> |
| Pharmaceuticals and Medical Devices Agency (JP) | <https://www.pmda.go.jp/> |
| Ministry of Food and Drug Safety (KR) | <https://www.mfds.go.kr/eng/index.do> |
| MHRA Public Access Registration Database (UK) | <https://pard.mhra.gov.uk/> |
| National Institute for Health and Care Excellence (UK) | <https://www.nice.org.uk/> |
| Food and Drug Administration (US) | <https://www.fda.gov/> |

**Supplementary table 2 A list of regulatory authorities for review reports**

| **Regulatory authority (Country)** | **Link** |
| --- | --- |
| DiGA directory (DE) | <https://diga.bfarm.de/de/verzeichnis> |
| Pharmaceuticals and Medical Devices Agency (JP) | <https://www.pmda.go.jp/PmdaSearch/kikiSearch/> |
| National Institute for Health and Care Excellence (UK) | <https://www.nice.org.uk/guidance/conditions-and-diseases> |
| Food and Drug Administration (US) | 501(k):<https://www.accessdata.fda.gov/scripts/cdrh/cfdocs/cfpmn/pmn.cfm>  De Novo: <https://www.accessdata.fda.gov/scripts/cdrh/cfdocs/cfPMN/denovo.cfm>  PMAs: <https://www.accessdata.fda.gov/scripts/cdrh/cfdocs/cfPMA/pma.cfm> |

**Supplementary table 3 A list of clinical trial registration systems**

| **Clinical trial registration system** | **Link** |
| --- | --- |
| Clinicaltrials.gov | <https://clinicaltrials.gov/> |
| Clinical Research Information Service | <https://cris.nih.go.kr/cris/search/listDetail.do> |
| International Clinical Trials Registry Platform | <https://trialsearch.who.int/> |
| International Standard Randomized Controlled Trial Number Registry | <https://www.isrctn.com/> |
| Japan Registry of Clinical Trials | <https://jrct.niph.go.jp/> |
